# Dataset: A consolidated and harmonised Verbal Autopsy dataset from Health and Demographic Surveillance Sites in South Africa

**DOI:** 10.12688/f1000research.55377.1

**Published:** 2023-05-19

**Authors:** Eilidh Cowan, Lucia D'Ambruoso, Jessica Price, Edward Fottrell, Kobus Herbst

**Affiliations:** 1 School of Geosciences, University of Edinburgh, Edinburgh, UK; 2 Aberdeen Centre of Health Data Science (ACHDS), Institute of Applied Health Sciences, School of Medicine, Medical Sciences and Nutrition,, University of Aberdeen, Aberdeen, UK; 3 Department of Epidemiology and Global Health, Umea University, Umea, Sweden; 4 4. MRC/Wits Rural Public Health and Health Transitions Research Unit (Agincourt), School of Public Health, Faculty of Health Sciences,, University of the Witwatersrand, Johannesburg, South Africa; 5 National Health Service, Grampian, UK; 6 Institute for Global Health, University College London, London, UK; 7 Africa Health Research Institute, Johannesburg, South Africa; 8 DSI-MRC South African Population Research Infrastructure Network (SAPRIN), Johannesburg, South Africa

**Keywords:** South Africa; Verbal Autopsy; Cause of death; Circumstances of Mortality

## Abstract

This data note provides details of the development of a Verbal Autopsy (VA) dataset produced with the South African Population Research Infrastructure Network (SAPRIN) drawing on datasets from health and socio-demographic surveillance sites’ (HDSS) ‘ covering a population of over 250,000 in two rural provinces in South Africa for the period 2012–2019. The purpose of the data set was to refine an analytical tool within VA, which provides unique information on care seeking and utilisation at and around the time of death complementary to that of medical cause of death. On an individual basis, the dataset includes demographic data, probable cause of death data, and data on care seeking and utilisation at or around the time of death drawn from longitudinal population cohorts. The purpose of this publication is to describe both the dataset and methods in formatting and processing the data for other researchers who may be interested in similar data. The data described in this paper are available to be requested from the respective HDSS repositories.

## Introduction

Every year, the medical causes of approximately 30 million deaths, half of all deaths worldwide, are not formally registered ^1^. These deaths occur predominantly in low- and middle-income countries where there is a lack of complete and functioning civil registration and vital statistics (CRVS) systems ^2^. Verbal autopsy (VA) is currently the only realistic alternative to medical certification of deaths in settings where CRVS is incomplete or absent. VA is a pragmatic survey-based method in which trained fieldworkers gather information from final caregivers on signs and symptoms of the deceased prior to death. VA data are then interpreted, by physicians or computer models, to determine probable cause(s) of death ^3^. The method is used to quantify levels and causes of death in otherwise unregistered populations. The World Health Organization (WHO) leads the development of international standards for VA.

This data note provides details of the development of a Verbal Autopsy dataset produced with the South African Population Research Infrastructure Network (SAPRIN) drawing on datasets from health and socio-demographic surveillance sites’ (HDSS. The purpose of the data set was to refine an analytical tool within VA, which provides unique information on care seeking and utilisation at and around the time of death complementary to that of medical cause of death.

Acknowledging the social determinants of heath as the fundamental causes of avoidable mortality and health inequalities, we sought to develop a systematic and scalable categorization system for circumstantial drivers of deaths ^4^. We previously devised an approach within VA tools called Circumstances of Mortality Categories (COMCAT) ^5^. The system is designed for large scale population assessment of burden of disease inclusive of the needs and behaviours of individuals and the responsiveness of the health system towards these ^6^. For example, a woman whose cause of death is assigned as obstetric haemorrhage might have died at home, while another woman with the same cause of death might have been inadequately managed despite reaching a facility. Measuring these scenarios at population level will provide important information for health services and reducing avoidable mortality.

The development of the COMCAT model began with the supplementation of existing interview questions on medical causes of death, to include input questions on care seeking and utilisation at and around the time of death, which were taken up in the 2012 WHO VA standard ^7^. From this, models were developed within existing automated VA data interpretation tools to assign likelihoods to circumstantial categories for each death on: emergencies, recognition of illness severity, use of traditional medicine, accessing care, and perceptions of poor quality of care ^5^.

This paper describes the collation and formatting of a mortality dataset from Health and Demographic Surveillance Sites (HDSS) in South Africa for use in refining the COMCAT system. HDSS are geographically defined populations that undergo continuous demographic monitoring. All vital events, such as births and deaths, are regularly recorded to track population change and highlight health and social care priorities ^8^. The dataset harmonises and links routinely collected VA data from the South African Population Research Infrastructure Network (SAPRIN). SAPRIN is a national research infrastructure funded by the National Department of Science and Innovation that aims to harmonise and integrate South Africa’s HDSSs.

## Methods

Each HDSS had a specific VA questionnaire that, since 2012, is broadly based on the WHO-2012 or WHO-2016 standard. VA data are collected electronically at household level by trained fieldworkers. Trained fieldworkers select responses to the questions from a specified set of answers, with logical skips and validation rules consistent with the WHO standard. Data quality control is carried out on al captured questionairres by specific HDSS team supervisors using either RedCap or Survey Solutions. We obtained all VA data, from the three HDSS’ included in the SAPRIN Network that had been collected on deaths that occurred from 2012 onwards. This was in order to increase the likelihood of inclusion of the COMCAT data, which were included in the WHO standard since 2012.

As each HDSS has a unique VA questionnaire, we aligned each of the HDSS’ questionnaires and potential responses to the WHO-2016 standard. As the VA interpretation tools are based on the WHO standard, in doing this we ensured the required indicators were available to utilise both a VA data formatting packages (PyCrossVA) and one of the automated VA interpretation tools to generate probable cause of death. A common data specification was developed that would retain maximum information but allow us to utilise one of the VA interpretation tools. VA interpretation tools use mathematical formulae, such as Bayes theorem, to calculate the probability of cause of death from a prior set of probabilities relating to input indicators, from the VA questionnaire ^9^.

After formation of the data specification, data were examined, as detailed above, to ensure the dataset included the indicators required to be processed in a VA interpretation tool to output both a reliable probable cause of death and COMCATs. A variety of additional indicators to the WHO standard had been included in the different sites’ questionnaires. These indicators were not included in the consolidated dataset as they are not required for the automated VA tool. However, individual case ID remained consistent throughout and these additional indicators could be included from the original dataset if of interest after the data had been processed by the VA interpretation tool. At this stage, we excluded one of the HDSSs, DIMAMO, as they did not have relevant data on the COMCAT input indicators. Data were then recoded and renamed in line with the newly developed data specification, this was done in pyCrossVA, a Python package (Python Programming Language, RRID:SCR_008394) developed to format VA data from WHO standard into the format for use in the desired VA interpretation tools. At this stage, we processed the data using the InterVA-5.1 interpretation tool in R 3.61 (R Project for Statistical Computing, RRID:SCR_001905). InterVA-5 was selected as this is currently the only tool that will output COMCATs, and refining these was the objective for the use of the data.

At all stages, data were processed individually by HDSS’. After the data had been processed through InterVA-5.1 we then added an additional variable of HDSS name to allow us to differentiate these by location before appending the two datasets. The final data set included records of 7980 deaths, 5924 and 2056 from Agincourt and AHRI HDSS respectively, for the period of 2012–19, and consisted of 25 variables detailing, basic demographics, probable cause of death, COMCAT and COMCAT input indicators.

The data were subject to consistency checks in InterVA-5.1. These are carried out before probable causes of death are determined for each individual death, where possible errors will be adjusted by InterVA-5.1 using other questions. These generate warning messages that can be interpreted by researchers. For example, a record of a male that has identified as pregnant will generate a warning message and, depending on the other information available, one of these inputs (i.e. male or pregnant) will be deemed an error and corrected by InterVA-5.1. Further to this, we excluded those aged over 100 years due to the unreliability of the data given the average life expectancy in the region.

## Software availibility

Software packages used to both format and process VA data are all open source and are available from the following ‘ https://github.com/verbal-autopsy-software’. These packages also contain functions to analyse VA data. 

## Data availability statement

The data described in this study cannot be made available to the public in an open repository due to the sensitive nature of the data. However, the data are available to be requested from SAPRIN or the respective HDSS repositories. Requests for the data can be made at the following link https://saprindata.samrc.ac.za/index.php/catalog/33.
