# Supplementary material for: Dataset: A consolidated and harmonised Verbal Autopsy dataset from Health and Demographic Surveillance Sites in South Africa
Source: F1000Res. 2023 May 19;12:520. [Version 1] doi: 10.12688/f1000research.55377.1 (PMC11399759; doi:10.12688/f1000research.55377.1)
Supplement: Peer review history

# Reviewer report: Reviewer response for version 1

**DOI:** 10.5256/f1000research.58945.r174907

**Published:** 2024-09-13

**Authors:** Tom Smith

**Affiliations:** 1 Swiss Tropical and Public Health Institute, Basel, Switzerland

The article provides a well written description of the rationale for the dataset. If anything, the importance of this is understated, since analysis of such dataset is crucial for understanding how to reduce mortality across the world.

 Unfortunately the authors do not actually provide the data, stating instead that "the data are available to be requested from SAPRIN or the respective HDSS repositories", a statement which the reader must take on trust.

 I noted a few minor typos:

 (HDSS should read (HDSS).

 "The purpose of the data set was ... " should be "... is... " I think.

 Citations should be provided for the software used (e.g. Red Cap)

Are sufficient details of methods and materials provided to allow replication by others?

Partly

Is the rationale for creating the dataset(s) clearly described?

Yes

Are the datasets clearly presented in a useable and accessible format?

No

Are the protocols appropriate and is the work technically sound?

Yes

Reviewer Expertise:

Epidemiology

I confirm that I have read this submission and believe that I have an appropriate level of expertise to confirm that it is of an acceptable scientific standard, however I have significant reservations, as outlined above.

# Reviewer report: Reviewer response for version 1

**DOI:** 10.5256/f1000research.58945.r237763

**Published:** 2024-02-06

**Authors:** Bruno Masquelier

**Affiliations:** 1 Université Catholique de Louvain, Louvain, Belgium

I read this short article with interest, as it deals with an important subject. I agree with the authors on the importance of complementing traditional verbal autopsies with additional, standardized information on the circumstances surrounding death, regarding the social determinants of health and care-seeking. The COMCAT tool is a significant contribution in this respect. It is also important to acknowledge the heavy work done on standardizing and pooling together datasets from different HDSS. However, I don't quite see the point of this article specifically, as it doesn't provide results, it doesn't document a database in the public domain, and it doesn't specify how the consolidated database can be acquired, and under what conditions. The article looks more like a deliverable for a project, but the contribution to scientific literature is not obvious to me at this stage. I would invite the authors to provide a sample anonymized dataset, as suggested by another reviewer, or to detail the procedures required to access the dataset. More info on the variations in data quality or response rates across HDSS sites concerning the COMCAT questions would also strengthen the paper. 

 Minor comments

 - In the introduction, the authors mention that the cause of death of 30 million deaths is not formally recorded in a CRVS system. But they base this assertion on a 2007 article by Setel and colleagues. I imagine the situation has changed over the past 15 years, and more recent estimates are available.

 - In the "methods" section, the authors mention additional indicators and indicate that they could be associated with this database based on individual IDs. Could you give some examples?

 - small typo="al captured questionnaires"

Are sufficient details of methods and materials provided to allow replication by others?

Partly

Is the rationale for creating the dataset(s) clearly described?

Yes

Are the datasets clearly presented in a useable and accessible format?

No

Are the protocols appropriate and is the work technically sound?

Yes

Reviewer Expertise:

Demography, child and adult mortality estimation

I confirm that I have read this submission and believe that I have an appropriate level of expertise to confirm that it is of an acceptable scientific standard, however I have significant reservations, as outlined above.

# Reviewer report: Reviewer response for version 1

**DOI:** 10.5256/f1000research.58945.r174908

**Published:** 2023-06-21

**Authors:** Tathagata Bhattacharjee

**Affiliations:** 1 Department of Population Health, London School of Hygiene and Tropical Medicine, London, UK

The paper brings out an important aspect for the need to prepare datasets for VA analysis. The method is crisply explained. However, sharing a sample anonymized dataset would have been more appreciated along with some sample code implementations for more clarity on the implementation which would help researchers to replicate or derive guidance on the processes in preparation of such datasets.

Are sufficient details of methods and materials provided to allow replication by others?

Yes

Is the rationale for creating the dataset(s) clearly described?

Yes

Are the datasets clearly presented in a useable and accessible format?

Yes

Are the protocols appropriate and is the work technically sound?

Yes

Reviewer Expertise:

data integration, ETL, record linkage, data standardization, OMOP /OHDSI

I confirm that I have read this submission and believe that I have an appropriate level of expertise to confirm that it is of an acceptable scientific standard.
